# Inflammation, Fibrosis and Cancer: Mechanisms, Therapeutic Options and Challenges

**DOI:** 10.3390/cancers14030552

**Published:** 2022-01-22

**Authors:** Bocheng Wu, Quaovi H. Sodji, Adegboyega K. Oyelere

**Affiliations:** 1 School of Chemistry and Biochemistry, Georgia Institute of Technology, Atlanta, GA 30332, USA; bocheng.wu@gatech.edu; 2 Department of Radiation Oncology, Stanford University, Stanford, CA 94305, USA; qsodji@stanford.edu; 3 Parker H. Petit Institute for Bioengineering and Bioscience, Georgia Institute of Technology, Atlanta, GA 30332, USA

**Keywords:** tissue inflammation, fibrosis, idiopathic pulmonary fibrosis, cystic fibrosis, hepatocirrhosis, interstitial lung disease, cancer, hepatocellular carcinoma, TGF-β pathway, NF-κB pathway, STAT3 pathway, COX/LOX pathway, epigenetic regulation, HDACI, immunotherapy, combinational treatment, tissue targeting, prodrug, nanoparticle delivery, structure–activity relationship study

## Abstract

**Simple Summary:**

Inflammation is like the proverbial double-edged sword. Controlled short-term inflammation could be a protective response while uncontrolled inflammation sustains multiple disease types, including several chronic diseases and cancers. The aim of this review article was to provide an in-depth analysis of the relationships and distinctions between uncontrolled inflammation, fibrosis and cancers. Herein, we emphasized the challenges and opportunities of developing novel therapies for the treatment and/or management of these diseases. We described how the therapeutic benefits of current agents could be enhanced through drug delivery systems, combination therapy and the integration of tissue-targeted and/or pathways selective strategies. In the pursuit of the next generation of safer therapies, we also postulated on the value of the re-evaluation of the disease-specific roles of multiple pathways implicated in the development and sustenance of chronic inflammatory diseases and cancers as well as the application of single-cell screening technologies in the discovery of novel disease-relevant proteins for the more precise targeting of these diseases.

**Abstract:**

Uncontrolled inflammation is a salient factor in multiple chronic inflammatory diseases and cancers. In this review, we provided an in-depth analysis of the relationships and distinctions between uncontrolled inflammation, fibrosis and cancers, while emphasizing the challenges and opportunities of developing novel therapies for the treatment and/or management of these diseases. We described how drug delivery systems, combination therapy and the integration of tissue-targeted and/or pathways selective strategies could overcome the challenges of current agents for managing and/or treating chronic inflammatory diseases and cancers. We also recognized the value of the re-evaluation of the disease-specific roles of multiple pathways implicated in the pathophysiology of chronic inflammatory diseases and cancers—as well as the application of data from single-cell RNA sequencing in the success of future drug discovery endeavors.

## 1. Introduction

Uncontrolled inflammation is a salient factor in multiple disease types, including several chronic diseases and cancers. Inflammation can be directly linked to tissue injury, fibrosis and necrosis, and it can be life threatening [1,2]. In fact, the treatment of tissue fibrosis remains an unsolved medical challenge as there are currently no tools to effectively overturn the progression of tissue fibrosis and necrosis. Idiopathic pulmonary fibrosis, an example of tissue inflammation, remains unsolvable and irreversible once diagnosed, with a 5-year survival rate, which is lower than many types of cancer [3]. A direct connection between inflammation and cancer is that tissue injury, and the concomitantly produced inflammatory factors, promote cancer cell growth via dysfunction in chemokine and cytokine signaling [4]. It is known that some cancer types rely on inflammatory signals for their progression, angiogenesis, proliferation and survival, invasiveness and metastasis [5,6,7]. Therefore, targeting inflammation is a promising therapeutic option for those types of cancers. However, challenges including low potency, poor drug distribution at disease sites and off-target effects, which result in overt toxicity, remain unsolved in anti-inflammation and anti-cancer drug development. Several comprehensive reviews that have probed the link between inflammation and cancer, cancer and fibrosis and inflammation and tissue fibrosis have appeared in recent studies [8,9,10,11,12,13,14,15,16,17]. This review describes the relationships and distinctions between uncontrolled inflammation, fibrosis and cancer while emphasizing the challenges and opportunities of developing novel therapies for the treatment and/or management of these diseases.

## 2. Tissue Inflammation

Inflammation involves the upregulation of pro-inflammatory signals, and it serves dual roles in biology. Controlled inflammation can protect against infection and tissue injury while uncontrolled inflammation sustains autoimmunity and malignant transformations [18]. Reactive oxidative species (ROS) produced in response to tissue injury, bacteria or virus infections, chemical stimulation, hypoxia, or within the tumor microenvironment (TME) can stimulate tissue epithelial cells [19]. The stimulated epithelial cells further release ROS and pro-inflammatory cytokines to trigger the immune system response [20]. Pro-inflammatory cytokines, which include but are not limited to TGF-β, TNF-α, IL-1, IL-6, IL-8, IL-10 [21], bind to their respective receptors and cause pro-inflammatory signals within the cell. These signals may induce the activation of inflammation pathways such as TGF-β, STAT3, NF-κB, Hippo, arachidonic acid and epigenetic pathways. These pathways could promote the expression of different proteins to either resolve the inflammation, or perpetuate it and cause chronic injury, tissue fibrosis or tumorigenic transformation [22]. In some cases, the expressed proteins could be components of the extracellular matrix (ECM) that is supposed to heal and repair the tissue [23]. However, due to excess tissue damage or injury, ECM production could be out of control [24]. The accumulation of ECM causes tissue stiffness, necrosis and loss of function. In other cases, transcription factors could be activated, and pro-inflammatory proteins and cytokines may be expressed. Cytotoxic immune cells could be activated, resulting in an acute increase in tissue inflammation [25,26,27]. Proteins such as anti-apoptotic STAT and Bcl-2 family proteins could be activated with the cytokine storms that are produced by inflammation. These proteins are oncogenic proteins that could induce anti-apoptosis in cells and promote cell proliferation [28] through the activation of pathways such as the NF-κB pathway and STAT3 or the upregulation of histone deacetylases (HDACs) activities, resulting in the inhibition of tumor suppressors and the promotion of cell proliferation [29]. Therefore, inflammation from tissue/cell damage caused by injuries, infections and other stimuli may be related to tissue fibrosis and tumor progression.

### 2.1. Inflammation and Tissue Fibrosis

Fibrosis is the thickening and scarring of the connective tissues in organs, which could be induced by injuries from environmental stimuli, biohazards exposure, radiation, infection, genetic mutation or other unclear intracellular dysfunctions. The abnormally stressed microenvironment of the tissue causes the upregulation of cytokines to modulate stages of inflammation. The immune response is triggered to protect the tissue from further attacks by pathogens such as bacteria and viruses. However, this process is upregulated with chronic injuries, such as with liver cirrhosis triggered by HCV infection [30]. Due to continuous infection and inflammation, the immune system response augments the process of healing, resulting in the subsequent deposition of ECM and further deterioration with sustained inflammation and liver fibrosis.

#### 2.1.1. Idiopathic Pulmonary Fibrosis (IPF)

**Figure 1:**
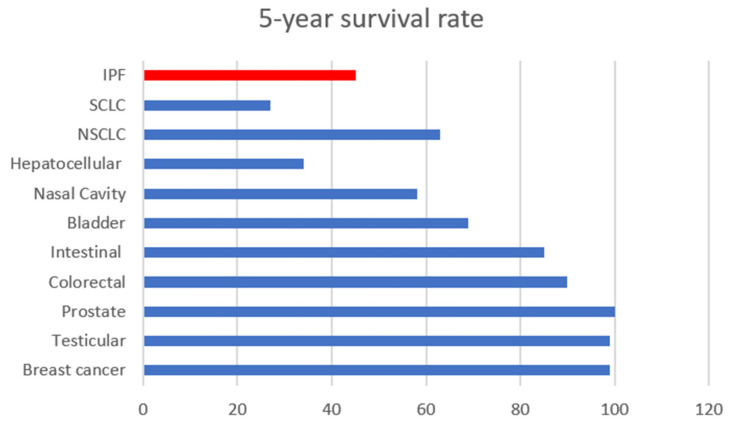
The 5-year survival percentage of idiopathic pulmonary fibrosis (IPF) relative to several types of cancers. The cancer 5-year survival data were retrieved from the American Cancer Society [37].

IPF is the most common and severe type of interstitial lung disease (ILD) [31]. IPF is a chronic and fatal disease that progressively decreases lung function. The causes of IPF are ambiguous, and it affects 5 per 10,000 people worldwide [32]. In the US, the mortality of IPF was 72.2 per 1,000,000 in 2017, which had remained stable since 2004 [33,34]. The median survival is approximately 2–5 years, with a 5-year survival rate of approximately 45% (Figure 1) [35,36].

**Figure 2:**
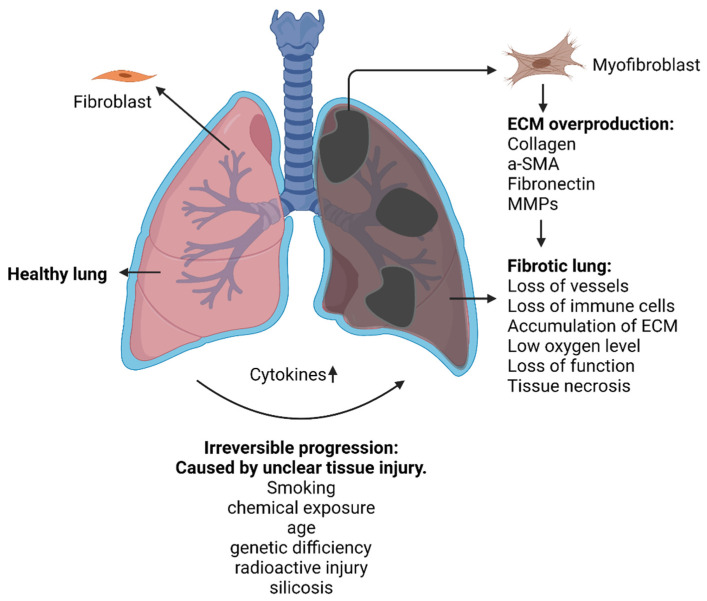
An illustration of extracellular matrix (ECM) overproduction in idiopathic pulmonary fibrosis (IPF).

IPF is characterized by the excessive accumulation of ECM components. Fibronectin, tenascin-C, α-smooth muscle cells (α-SMA) as well as collagen type I and III are secreted by fibroblasts in the process of fibrosis [38,39,40]. Fibroblasts, the most common cell to produce ECM, are activated by cytokines, growth factors and fibrotic factors to continuously generate ECM (Figure 2).

During IPF, several cells secrete cytokines to activate fibroblasts to become myofibroblasts which are key cells that overregulate ECM remodeling through a combination of synthesizing features of fibroblasts with cytoskeletal contractile characteristics of α-SMA [41]. During this process, other cell types are also involved. Specifically, during injury, epithelial cells undergo epithelial–mesenchymal transition (EMT) driven by pro-inflammatory factors such as TGF-$\beta_{1}$ [42], Wnt/β-catenin [43], epidermal growth factor (EGF) [44], connective tissue growth factor (CTGF) [45], fibroblast growth factor (FGF) [46] and nuclear factor-kB (NF-kB) [47]. The EMT transition regulates ZEB1 and Snail transcription factors and induces fibroblasts’ transformation into myoblasts, which enhances the production of the extracellular matrix. Therefore, EMT is one of the major sources of fibrosis, as it supplies more fibroblasts and supports the subsequent ECM production.

Lymphocytes could also induce the activation of fibroblasts via the T-cell helper 2 (Th2) polarized response. The Th2 response is an immune response against intestinal helminths and other extracellular injuries. Th2 polarization is induced by adhesion between endothelial cells and lymphocytes. This response leads to the secretion of pro-inflammatory cytokines such as IL-4, IL-5, IL-6, IL-10 and IL-13 which activate the fibroblasts and their transformation into myofibroblasts [48]. The next important activation of fibroblasts is through macrophages. There are two major sources of macrophages: those derived from the early embryo and those derived from monocytes [49,50]. The monocytes could be polarized into M1 or M2 macrophages after certain microenvironment cues [51]. In IPF, M2 is the major phenotype of macrophage which, as a result of the cytokines paracrine (IL-4, IL-10, IL-13 and TGF-β) of injured endothelial cell to the monocytes, stimulates tissue repair. [52]. However, the M2 phenotype over-stimulates the expression of the TGF-β signal, CTGF, platelet-derived growth factor (PDGF), EGF and IL-1α, which continuously activate the fibroblasts and induce tissue fibrosis [53]. On the other hand, M1, the pro-inflammatory macrophage phenotype, is usually regarded as anti-fibrotic in IPF. However, the M1 phenotype could exacerbate the inflammatory status of the lung injury and may trigger the process of fibrosis in IPF patients through the TLR4 and NF-κB pathway [53]. Thus, the balance of M1/M2 polarization is essential for the progression of IPF, and the regulation of polarization could be a key option for IPF treatment.

Based on the aforementioned literature observations, the main pathway towards IPF is the activation and proliferation of fibroblasts, as ECM components can only be over-expressed when myofibroblasts are formed. Thus, the inhibition of the activation and proliferation of fibroblasts is an effective strategy for preventing or delaying fibrosis progression.

#### 2.1.2. Liver Cirrhosis

**Figure 3:**
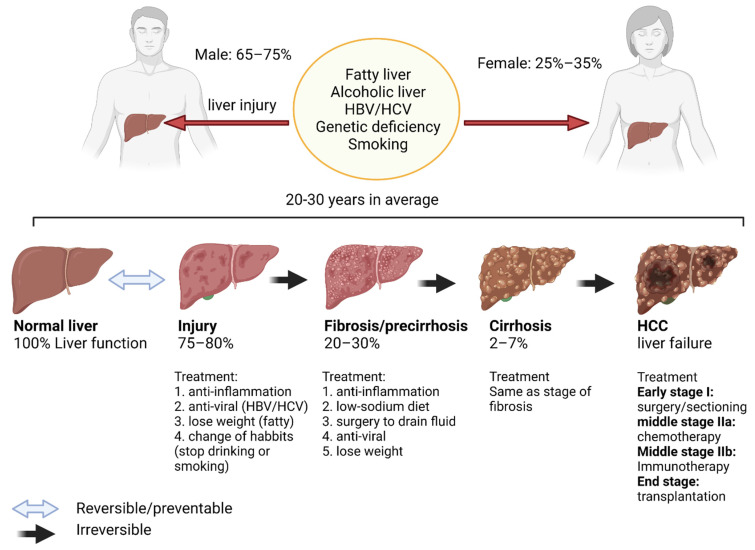
Progression from liver cirrhosis to hepatocellular carcinoma (HCC).

Similarly to IPF, inflammation is closely linked to the etiology of hepatocirrhosis (liver cirrhosis). Unlike IPF, however, the causes of liver cirrhosis are known and include alcoholic liver, fatty liver and HBV/HCV infections (Figure 3). Unfortunately, liver cirrhosis is irreversible and may progress to liver cancer and death. Globally, the number of deaths related to cirrhosis has increased from 1990 to 2017, counting 883,000 male deaths and 440,000 female deaths in 2017. Although this death rate has since been decreasing due to improvements in treatment, it remains elevated [54].

There are three stages of this disease: pre-cirrhotic disease, compensate cirrhosis and decompensated cirrhosis. In the pre-cirrhotic disease stage, major and continued inflammation and liver injury occur. Cytokine storms triggered by acute injuries, pathogens, cancers and autoimmune conditions cause the hyperactivation of immune cells. The prevention of further progression could be achieved with early treatments targeting the cause of injury. However, once the liver enters the next stage, fibrosis occurs to compensate cirrhosis and cannot be reversed. With the dysregulation in ECM production that is caused by pro-inflammatory intracellular signals, the accumulation of collagen, MMPs, α-SMA and fibronectin, liver functions are impaired. Treatments, including those with anti-fibrotic and anti-inflammation agents, can only delay further progression. During the decompensated stage, most liver functions, including detoxification and glucose metabolism, are significantly impacted due to fibrosis and necrosis. Patients with decompensated cirrhosis are considered for liver transplantation as no other curative therapy is available. Liver cancers such as hepatocellular carcinoma (HCC) may develop in up to 29.7% of cirrhotics patients within ten years [55]. Treatment options for HCC include radiotherapy, trans-arterial chemoembolization (TACE) and radiofrequency ablation (RFA). Other treatment options include immunotherapy with immune checkpoint inhibitors such as pembrolizumab. However, only 20% of patients respond to this therapy [56].

#### 2.1.3. Cystic Fibrosis

**Figure 4:**
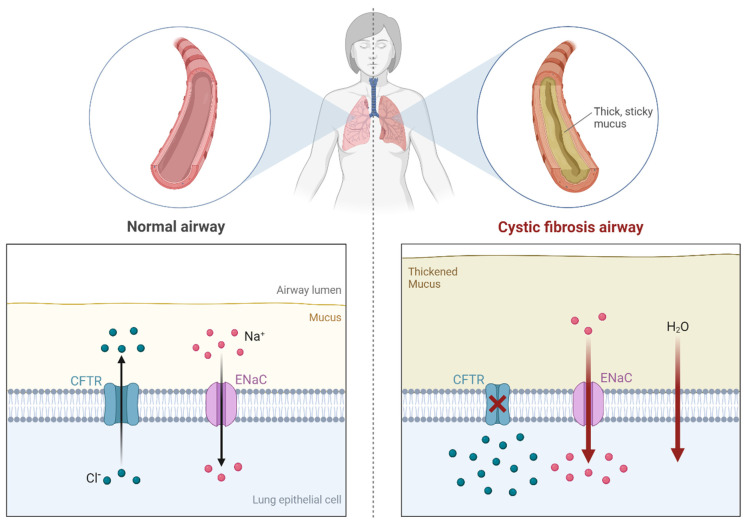
Illustration of the molecular basis of cystic fibrosis pathogenesis [61].

Unlike IPF or liver cirrhosis, cystic fibrosis (CF) is an autosomal recessive genetic disease associated with excessive and chronic inflammation of the airways due to infectious pathogens. In the United States (US), this disease is more common among the Caucasian population (1 in 2500–3500 newborns) relative to other ethnicities [57]. In most cases, CF is caused by the genetic mutation of the cystic fibrosis transmembrane conductance regulator (CFTR) gene (Figure 4). The result of the mutation is that the salt and fluid absorption by gland duct epithelia is effectively prevented, causing tissue inflammation in the respiratory system and other organs. Excessive mucus excretion takes place in these organs, which concomitantly thickens the tissues in these organs, leading to the blockage of normal fluids and the weakening of the immune system. In fact, chronic respiratory system bacterial infection and inflammation are the primary causes of CF morbidity and fatality [58]. A therapeutic option available to CF patients is the transplantation of damaged organs, especially of the lungs. According to recent estimates, more than 80% of children could survive 1 year after lung transplantation, 65% at 3 years, 54% at 5 years and 32% at 10 years [59]. In 2016, CF patients in the US had an average life expectancy of 47.7 years. However, the CF patients’ life expectancy has been on an increasing trend, largely due to new medications and therapy options [60].

The mucus-thinner medication Dornase Alfa, together with hypertonic saline and antibiotics, is the front-line treatment for CF patients. A gene-targeting tritherapy combining elexacaftor, tezacaftor and ivacaftor, known as Trikafta, is a new treatment approved in 2019, for CF with F508del mutation [62,63].

For multiple reasons, CF increases the risks of developing malignancy. Factors predisposing CF patients to cancer include the immunosuppressive therapies used to reduce immune rejection after organ transplantation [62,63,64]. Indeed, during a 20-year observational study of CF patients, Maisonneuve et al. found an increased risk of malignancy, especially that of cancers of the reproductive organs, kidneys and digestive tract such as esophageal, gastric, bowel and hepatic cancers after organ transplantation [65]. It is important to emphasize that immunosuppressive therapy increases the risk of malignancy in all transplanted patients, including those with CF. However, non-transplanted CF patients are at an increased risk of developing cancer, especially of the digestive tract organs, suggesting that other factors such as the chronic inflammation associated with continuous bacterial infections predispose CF patients to developing cancer [65]. Another study indicates that cystic fibrosis is correlated to liver cirrhosis (30% of CF patients) which ultimately leads to liver cancer [66,67].

#### 2.1.4. Drug-Induced Pulmonary Fibrosis

In addition to the aforementioned etiologies of tissue fibrosis, drugs also constitute an important cause of tissue fibrosis, especially in the lung. Over 300 drugs have been reported to cause lung injury that may result in lung fibrosis. Broadly, these include chemotherapy drugs such as bleomycin and cyclophosphamide; cardiovascular drugs including amiodarone and angiotensin converting enzyme (ACE) inhibitors; antimicrobials such as isoniazid and amphotericin B; biological drugs such as trastuzumab and bevacizumab; and even anti-inflammatory drugs such as nonsteroidal anti-inflammatory (NSAID) agents and aspirin [68]. Although the mechanism of drug-induced fibrosis has not been completely elucidated, the insult caused by these agents produces an initial inflammation and a recovery phase characterized by either the normal restoration of tissue architecture or the deposition of fibrotic tissue [69,70].

#### 2.1.5. Radiation-Induced Fibrosis

It is estimated that 52% of all cancer patients will receive radiotherapy during the course of treatment, with 60% of these patients treated with curative intent [71,72]. Radiotherapy is associated with acute and late toxicities including tissue fibrosis. Immediately following exposure to radiation, inflammatory cytokines are released into the extracellular milieu, resulting in a cascade of events including microvascular changes and culminating in local inflammation [73]. The manifestations of radiation-induced fibrosis are based on the radiated field and organs present within such a field. For instance, radiation to the esophagus may result in strictures leading to decreased peristalsis [74], whereas patients with head and neck cancer treated with radiation therapy are at risk of developing trismus as a result of fibrosis and a contracture of mastication muscles which can impact the quality of life [75]. In breast cancer patients, treated with adjuvant radiation therapy after surgery, radiation-induced fibrosis can result in adverse cosmesis due to the retraction of breast tissue [76]. Radiation therapy for the treatment of lung cancer can also cause pulmonary fibrosis which can interfere with optimal gas exchange and pulmonary functions [77]. The factors contributing to the incidence of radiation-induced fibrosis can be dichotomized into treatment-related and patient-specific factors. Treatment-related factors include parameters such as the extent of the radiation field, dose and fractionation as well as the combination of multiple cancer treatment modalities such as surgery and chemotherapy with radiation, whereas patient-specific factors involve pre-existing comorbidities such as IPF and connective tissue diseases [78,79].

Although radiation-induced fibrosis is regarded as a long-term toxicity in patients undergoing radiotherapy, advances in radiation delivery such as image-guided radiotherapy (IGRT), intensity-modulated radiotherapy (IMRT) and tumor motion management techniques have contributed to the decreased incidence of short- and long-term toxicities associated with radiation therapy by minimizing radiation delivery to normal healthy tissue adjacent to the tumors [80]. Despites these aforementioned advances, radiation-induced fibrosis and the preceding inflammation remain challenges not only in patients with comorbidities such as IPF and connective tissue diseases but may also interfere with subsequent surveillance imaging after treatment completion in most patients. For instance, current imaging modalities such as computerized tomography (CT) or positron emission tomography (PET) used for surveillance after radiation therapy may have difficulty in distinguishing between the local recurrence of a tumor and radiation-induced changes including fibrosis, thus subjecting patients to unnecessary biopsy for diagnosis confirmation [81].

### 2.2. Inflammation and Cancer

According to clinical observations, cancers are highly associated with tissue inflammation. In addition to genomic and epigenetic drivers of tumorigenesis, the tissue microenvironment can also lead to cancer development and progression. As a consequence of sustained insults and attempts by the host immune system to resolve those events, immune cells such as macrophages, neutrophils and stromal cells such as fibroblasts secrete cytokines. However, these cytokines may act as a double-edge sword. While they attract immune cells in the microenvironment (chemokines) or lead to the proliferation of immune cells, cytokines can also enhance oncogenic transformation, promote metabolic changes and hypoxia, thus favoring tumorigenesis. The cytokine-induced inflammatory response whose initial goal is to resolve the underlying insult can promote tumorigenesis as various molecular pathways are common to inflammation and carcinogenesis response pathways [82]. Following tumorigenesis, malignant cells can also secrete tumor-promoting cytokines, exerting their actions through autocrine mechanisms. Furthermore, tumors can also induce tissue inflammation by secreting inflammatory cytokines in their microenvironment to maintain ideal tumor growth conditions. However, a key cytokine, whose function further demonstrates the crucial roles of inflammation in sustaining tumor growth, is IL-10, which inhibits the inflammatory effects of IL-6 and IL-12/IL-23 and inhibits tumor-promoting inflammation. It also enhances the cytotoxic activity of intratumoral CD8^+^ T-cells, as preclinical models have shown that the de novo expression of recombinant IL-10 results in CD8^+^ T-cell-mediated tumor rejection [83].

#### 2.2.1. Cancer Types Linked to Chronic Tissue Inflammation

Several studies have shown that the dysregulation of inflammatory signals caused by chronic injuries could progress into malignant transformation. For example, chronic liver tissue injury caused by hepatitis infection predisposes hepatitis patients to higher risks of cirrhosis and hepatocellular carcinoma (HCC). Colitis-associated cancer (CAC) is a subtype of colorectal cancer that is known to be associated with inflammatory bowel disease (IBD) [84]. Additionally, IPF patients also have higher risks of lung cancer [85,86]. Table 1 summarizes inflammation-associated cancer types and their inducers.

#### 2.2.2. Sources of Inflammation That May Lead to Cancers

Tissue inflammation is highly correlated to the development of several cancer types, as the initiation of cancers could be induced by the release of inflammatory cytokines and subsequent mutations ensuing from the numerous cycles of tissue damages and repairs. There are several sources of inflammation that could potentially trigger cancer. For example, sustained bacteria and virus infections could induce chronic tissue inflammation that may progress to cancer—while exposure to pro-inflammatory chemicals and stimulants may lead to specific cancers such as lung cancer. Additionally, abnormal immune attacks may trigger chronic inflammation that could lead to cancer.

##### Infections

Bacterial and viral infections are among the key causes of chronic tissue injuries. HCC is linked to liver cirrhosis that could result from infections by the hepatitis virus, including hepatitis B virus (HBV) and hepatitis C virus (HCV). The cytokines (TGF-β and IL-6) released as a result of these infections activate the hepatocyte stellate cells (HSCs), triggering immune response and inflammation [88]. Additionally, the failure of anti-viral treatment could induce chronic inflammation and liver tissue fibrosis in response to the overexpression of supposedly protective TGF-β. These events ultimately resulted in cell plasticity, tissue fibrosis and even carcinogenesis [89,90]. The chronic inflammation-triggered ROS production is the main cause of genetic mutation, which is responsible for a carcinogenic event taking place [91].

Recent studies found that the *Helicobacter pylori* (*H. pylori*) is the key pathogenic factor for chronic gastric inflammation. *H. pylori* infects 50% of the world population and its infection can cause duodenal and gastric ulcers [92]. *H. pylori*-infected cells release IL-8, IL-10 and TNF-α which directly stimulate the immune cells in the microenvironment, leading to tissue inflammation such as gastritis. In addition to other factors, such as smoking and high salt consumption diet, chronic inflammation caused by *H. pylori* infection can also induce gastric cancer [93].

##### Chemical Exposure

Exposure to chemicals is another inducer of chronic inflammation. Lungs tissues are most susceptible to the effects of chemical exposure which could cause chronic injuries such as bronchitis and IPF. Smoking, silica exposure and the inhalation of hazardous chemicals are the major sources of irritants that can cause chronic lung tissues inflammation linked to lung cancer. The lung tissue injuries induced by these chemical irritants cause integrins αvβ6 and αvβ8 to stimulate and induce the overexpression of TGF-β [94]. The binding of TGF-β to TGF-βR in epithelial cells or fibroblasts causes EMT or myofibroblast differentiation [95,96] which induces an overexpression of ECM and a change of metabolism in cells—from aerobic glycolysis to anaerobic glycolysis—to provide the extra energy needed for ECM production and migration. Stiffness and lung tissue necrosis occur when ECM is overproduced, thus triggering tumorigenesis [8].

##### Autoimmune Conditions

Autoimmune conditions are diseases associated with the dysregulation of the immune system, resulting in the immune system attacking specific organs. The immune system attack may strongly induce inflammation and in some instances tumorigenesis [97]. Type-1 diabetes (T1D) is a classic example of an autoimmune disease characterized by the dysregulation of immune cells leading to the destruction of insulin-producing cells of the pancreas [98]. Additionally, T1D induces the chronic inflammation of the pancreatic islets of Langerhans. Such inflammation may ultimately progress into pancreatic cancer and perhaps other cancers [99]. Ulcerative colitis is another example of an autoimmune disease that induces colonic inflammation (such as inflammatory bowel disease), and in certain circumstances, ultimately develops into colon cancer [100].

Paradoxically, the long-term treatment of autoimmune diseases also increases the risk of cancer, because the use of immunosuppressive drugs provides a microenvironment suitable for cancer growth. It has been found that cyclosporine, an immunosuppressive drug for managing several autoimmune diseases, can increase the risk of cancers such as skin cancers and lymphoma [101].

## 3. Major Inflammatory Pathways in Tumor Progression and Tissue Fibrosis

Burgeoning literature data have evidenced an association between inflammation and tumorigenesis. Specifically, it is clear that cytokines and chemokines released during inflammation may induce mutation in cells. However, the mechanisms at the cellular level remain complicated. Herein, we will discuss six major cellular pathways, the potential treatment options that target these pathways and their current challenges.

### 3.1. TGF-β Pathway and Inflammation-Induced Carcinogenesis

**Figure 5:**
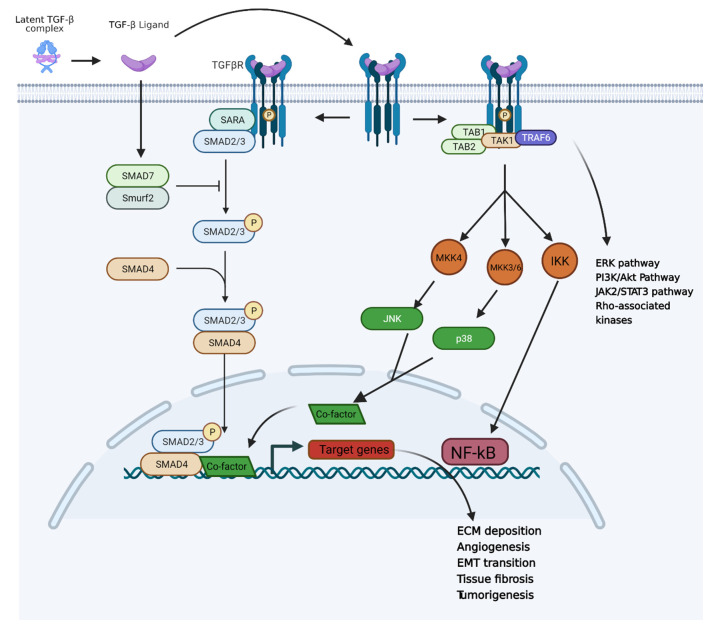
TGF-β pathway in cancer and inflammation [117].

According to our current understanding, TGF-β plays the role of a double-edged sword in inflammation and cancer. In the case of common acute injury, TGF-β is an important anti-inflammatory cytokine that can protect tissue from injury. It also plays an essential role in tissue repair. However, TGF-β could also be a pro-inflammatory cytokine under the conditions of a chronic injury and a tumor microenvironment. The activation of TGF-β is usually caused by tissue inflammation, ROS upregulation and immune response. TGF-β has three isoforms, which are TGF-β1–3, among which TGF-β1 plays the critical role of inducing inflammation [102]. TGF-β is often secreted by cells in a large latent complex (LLC) in which the TGF-β is protected by the latency-associated peptide (LAP) and covalently bond to a family member of the “latent TGF-β-binding proteins” (LTBPs) [103,104]. Upon cleavage, the small latent complex (SLC) will be formed. However, TGF-β needs to be activated via the release of LAP. Only a few cell types can secret the SLC form of TGF-β. Additionally, the SLC form can be activated by non-integrin activation [104], including low pH (pH < 4) [105], protease activity (MMPs) [106], ROS [107] and thrombospondin-1 [108] (TSP-1)-induced LAP transformation. Integrins αvβ6 and αvβ8 play roles in TGF-β activation via different mechanisms. Specifically, the LLC form of TGF-β binds to αvβ6 or αvβ8 of the other cell on the membrane. For αvβ6, cell traction and pulling induce conformational change in LAP to release TGF-β in its active form. Integrin αvβ8 induces MT-1 MMP into the LAP interaction, and therefore a proteolytic cleavage between LAP and TGF-β [109]. The released TGF-β binds to TGF-βRs to induce inflammation pathways. With TGF-β ligand–receptor binding, the SMAD2/3, PI3K, JNK and MAPK pathways could be activated. The SMAD pathway activation induces pro-inflammatory cytokines and ECM production [110]. The activated PI3K pathway induces cell proliferation and invasiveness, while the JNK and MAPK pathways induce cell stress, inflammation and tissue necrosis [111]. The TGF-β activation and overregulation could also induce EMT through the transcriptional regulation and expression of Snail families, ZEB families and bHLH families [112], which could induce malignant tumor growth [42,113]. In addition, angiogenesis could also be promoted by TGF-β through the expression of ID-1 and ID-3 in terms of SMADs transcriptional regulation [114,115,116]. Overall, the TGF-β pathway is central to the connection between chronic inflammation and tumorigenesis (Figure 5).

The acidic tumor microenvironment promotes TGF-β activation that assists in building a fibrotic environment around the tumor. Within the microenvironment, cancer-associated fibroblasts (CSFs) are the major producer of TGF-β cytokines [118] that will stimulate excessive ECM deposition or remodel the ECMs and form desmoplasia, a term to indicate the fibrosis microenvironment around the tumor. Desmoplastic reaction shields the tumor from exposure to chemotherapeutic agents and significantly increases tumor growth, angiogenesis, as well as promotes cancer cell invasiveness and metastasis [119]. Pancreatic and triple negative breast cancers are especially prone to desmoplasia formation. Once desmoplasia is built, it is hard to treat the cancer with small molecule drugs [120,121].

**Figure 6:**
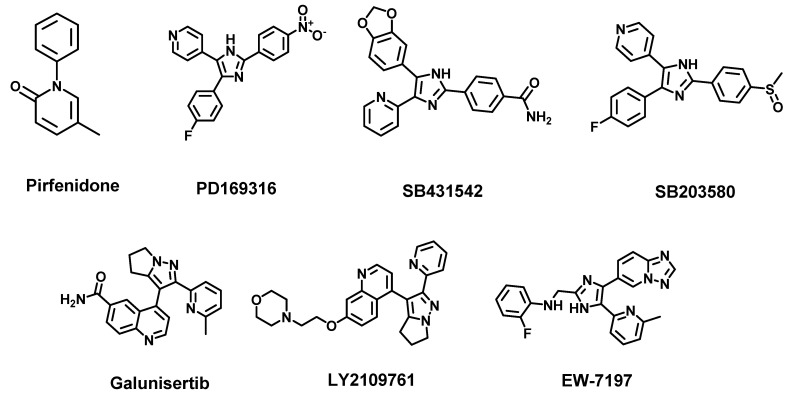
Structures of representative examples of small molecule inhibitors of the TGF-β pathway.

TGF-β pathway inhibition is a promising strategy for managing/treating fibrosis, inflammation and cancer. Therapeutic targets within the TGF-β pathway include integrin, TGF-βR, TGF-βR kinase, SMAD2/3 phosphorylation and TGF-β expression. The anti-integrin antibody can block integrin αvβ6 binding with LAP-TGF-β and thus prevent the release of activated TGF-β. On the other hand, the MT-1 MMP antibody can also effectively reduce the activation of TGF-β by inhibiting the function of αvβ8. The strategies to inhibit TGF-βR include ligand traps, TGF-βRI kinase inhibitor and TGF-βRII kinase inhibitors. For the TGF-β ligand trap, small molecule drugs such as pirfenidone (PFD) (Figure 6) and its derivatives were found to be ligands for the receptor. PFD has been approved by the FDA for the treatment of IPF [122,123]. In addition, PFD is also known to be a potential anti-tumor agent due to its TGF-β pathway inhibition [124]. Peptide probes, which have demonstrated selective inhibition effects for integrin and TGF-βRs, have been reported [125,126]. For kinase inhibition, selective TGF-βR kinase inhibitors such as SB431542 (Figure 6) have been shown to potently inhibit both cancer cell growth and inflammation. On the other hand, the inhibition of p38 and SMAD phosphorylation, which suppresses TGF-β upregulation, has been achieved with small molecule inhibitors PD169316 and SB203580 (Figure 6) [127].

### 3.2. TNF-Alpha, NF-κB Pathway and Inflammation

**Figure 7:**
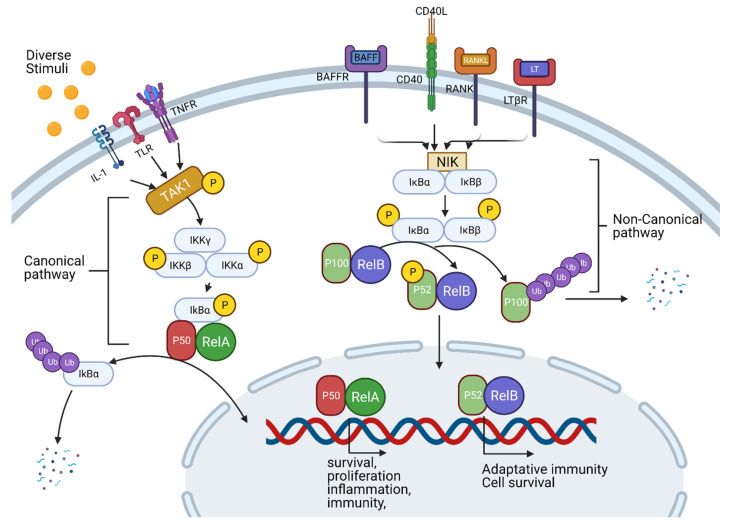
Cell signaling and pathways of NF-κB-mediated inflammation.

The transcription factor NF-κB is a regulator of inflammation and immune response. The NF-κB pathway is crucial to the survival of several cancer types as it prevents cell death and promotes cell proliferation by inhibiting tumor suppressors such as p53. Several pro-inflammatory ligands and their receptors (cytokine receptors, pattern-recognition receptors (PRRs), TNF receptor and T-cell receptors) activate the NF-κB pathway [38]. It has been shown that NF-κB signaling has two separate pathways. The “canonical” pathway is stimulated by TNF-α and IL-1 or TLR to cause the induction of IκBα phosphorylation by IKK (Figure 7). IκBα is a protein associated with and inactivated by p50/Rel-A or p50/c-Rel dimers. The phosphorylation of IκBα promotes its degradation by the ubiquitin–proteasome degradation pathway; the consequently released Rel-A/p50 will translocate into the nucleus and promote the expression of pro-inflammatory cytokines, anti-apoptosis proteins, chemokines and cell cycle regulators. The downstream effect of this signaling cascade is the induction of tissue inflammation, necrosis or tumorigenesis. For the non-canonical NF-κB pathway, the stimulation from LTs, CD40L, or BAFF, causes IKKα activation and the release of the p100/Rel-B dimer into the nucleus (Figure 7). Both pathways are responsible for inflammation, tissue necrosis and tumorigenesis.

The upregulation of the NF-κB pathway in several cancer types promotes proliferation, invasiveness, metastasis and angiogenesis through the expression of NF-κB target genes [128]. For example, the expressions of Bcl-2 families of apoptosis regulators [129], caspase-8 inhibitor FLIP [130] and VEGF [131] are regulated by NF-κB. In addition, NF-κB also induces EMT, which directly contributes to cancer metastasis [132]. Thus, a targeted inhibition of NF-κB by pharmacological agents is a promising cancer therapy strategy.

**Figure 8:**
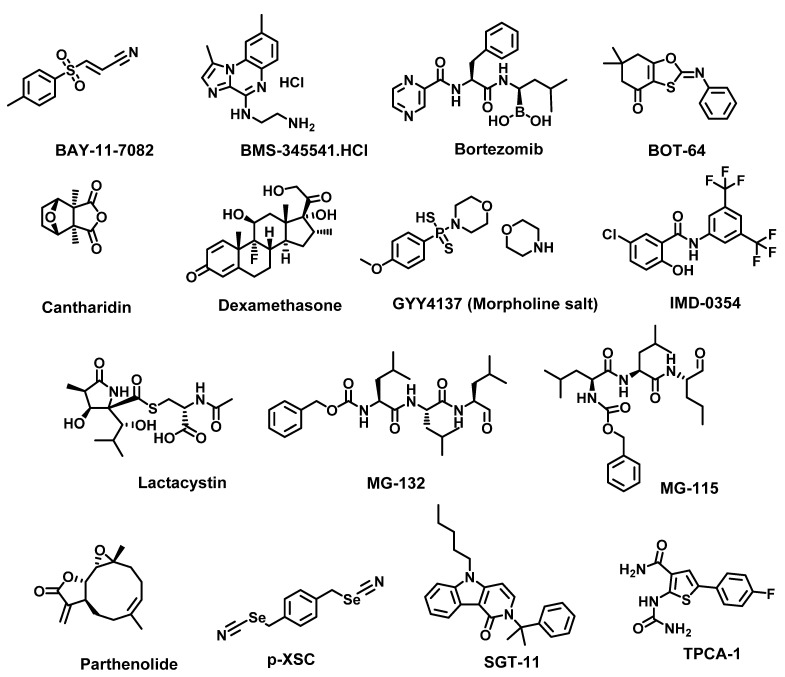
Structures of representative examples of small molecule inhibitors of the NF-κB pathway.

Approaches that have been investigated to achieve NF-κB inhibition include receptor inhibition, IKK complex inhibition, IκB degradation inhibitor, NF-κB DNA binding inhibitors, NF-κB translocation inhibitors, p53 induction, p65 acetylation and Nrf-2 activation. Receptor inhibitors such as TNFR inhibitor SGT-11 and IL-6R inhibitor Tocilizumab; IKK inhibitors such as TPCA 1 [133], BOT-64 [134], BMS 345541 [135] and IMD 0354 [136]; IκB degradation inhibitors such as BAY 11-7082 [137], parthenolide [138], lactacystin [139], MG-132 [140] and MG-115 [139,141]; NF-κB DNA binding inhibitors such as GYY 4137 [142] and p-XSC [143]; proteasome inhibitor bortezomib and natural product cantharidin (Figure 8) are all promising NF-κB pathway inhibitors [144]. In addition to these pathway-specific agents, the NF-κB pathway could also be inhibited by the corticosteroid dexamethasone [145] and anti-ROS agents such as lipoic acid [146] and dimethyl fumarate [147].

### 3.3. JAK-STAT Pathway and Inflammation

**Figure 9:**
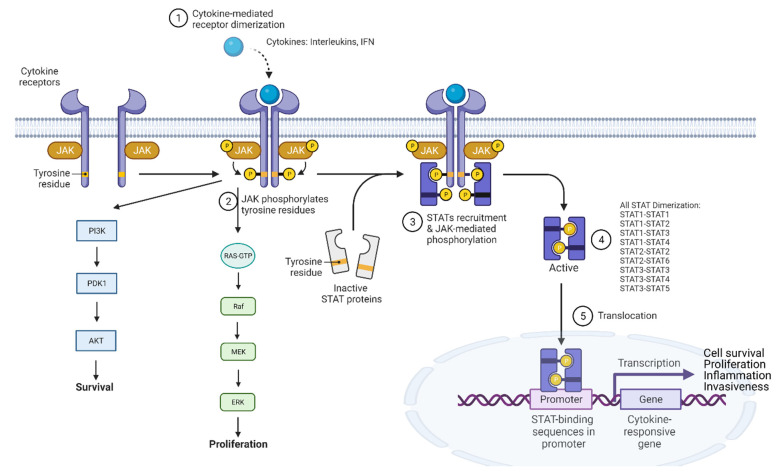
Generic intracellular STAT proteins signaling showing activation and STAT-target genes transcription.

Signal transducer and activator of transcription (STAT) is an essential regulator of inflammation signal in tissue inflammation. The STAT family consists of seven sub-members: STAT1, STAT2, STAT3, STAT4, STAT5a, STAT5b and STAT6. Each member plays a different role in regulating inflammation, proliferation, survival and tumorigenic activities [148]. In general, STAT proteins are activated through the receptors-ligands (interleukins, interferon, etc) interaction that stimulates the phosphorylation of Janus kinases (JAKs). The activated JAKs phosphorylate STAT; the dimerization of the phosphorylated STAT (p-STAT) results in the translocation of the p-STAT dimer into the nucleus where it induces the transcription of STAT-target genes (Figure 9).

The heterodimerization of STAT sub-members occurs in the cell and these heterodimers have different cellular activities (Table 2). Specifically, the STAT1–STAT2 dimer (as well as the STAT1–STAT1 and STAT2–STAT2 dimers) induces the transcription of pro-inflammatory and immunoregulation genes in interferon-stimulated cells in response to virus and bacterial infections [149,150]; the STAT1–STAT3 dimer induces cytokine production and inflammation or blocks the STAT1–STAT1 activity. On the other hand, STAT3–STAT3 dimerization induces cell proliferation, anti-apoptosis and invasion. STAT3–STAT3 dimerization (signaling) is upregulated in difficult-to-treat cancers such as the triple negative breast cancer (TNBC) [148] and HCC, as well as lung, breast, renal, ovarian cancers and lymphomas [151,152,153,154,155,156]. The STAT3–STAT5 heterodimer also promotes inflammation and tumorigenesis (to be discussed more below). Relative to others, STAT4 links to inflammation and cancer indirectly. Rather, it could be activated by IL-12, IL-2 IL-23, IL-32, IFN-1, IL-18, IL-21 [157] and help the maturation of T-cells into specialized T-cells by activating some genes, which connect with the activation of immune response and tissue inflammation. The known inflammatory diseases that can be linked to STAT4 include but are not limited to chronic hepatitis B (CHB), HCC, IBDs, HBV, systemic sclerosis (SSc) and type-1 Diabetes. In addition to promoting the production of pro-inflammatory cytokines, the STAT5–STAT5 dimer upregulates Akt proteins, p85 and p110 for the PI3K pathway to induce tumorigenesis and mutagenesis in several cancers including breast cancer, acute myeloid leukemia, prostate cancer and melanoma [158]. STAT3–STAT5 could act similarly to an inflammation promoter with cytokine activations [159] while the IL-4 and IL-13 activation of STAT6 promotes its involvement in inflammatory airway hyperresponsiveness [160], eosinophilic infiltration [161] and the responses of mast cells [162,163].

Among all STAT sub-members, STAT3 is the most important regulator of inflammatory factors that feed cancer progression. Specifically, the STAT3-mediated upregulation of anti-apoptotic proteins Bcl-2 families and Mcl-1 resists cell death, while its upregulation of cyclin D1 and c-Myc causes increased cell proliferation. Over-active p-STAT3 helps cancer cells evade the immune system through the upregulation of PD-L1 expression [164]. In addition, STAT3 enhances cancer cell directional migration by regulating Rac1 activity [165] while it promotes angiogenesis by transcriptionally regulating VEGF activity [166].

**Figure 10:**
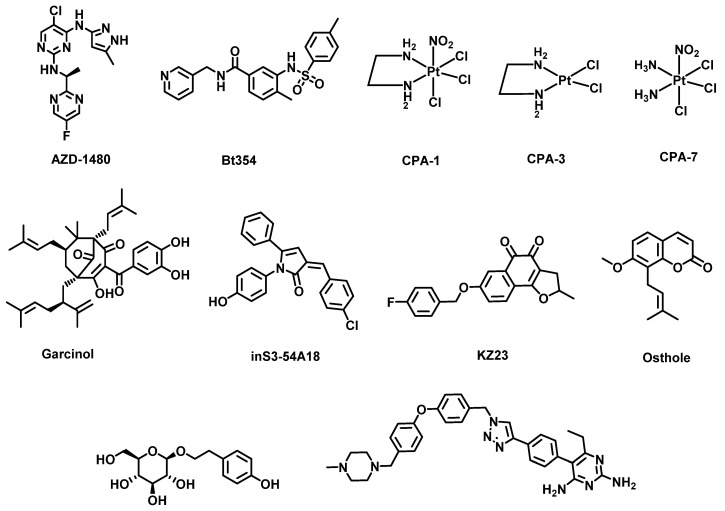
Structures of representative examples of STAT3 small molecule inhibitors.

Therapeutic agents targeting the STAT3 pathway (Figure 10) have been well studied as treatment modalities for cancers. STAT3 inhibitors investigated to date include JAK kinase inhibitors, STAT3 SH2 domain phosphorylation inhibitors, nuclear translocation inhibitors, DNA binding domain inhibitors and transcription inhibitors. Representative examples of small molecule STAT3 inhibitors are JAK kinase inhibitor AZD-1480 [166]; SH2 domain inhibitors Bt354 [167], osthole [168] and KYZ3 [169]; STAT3 translocation inhibitor farcinol [170]; STAT3 DBD inhibitor methylsulfonylmethane (as the VEGF promoter inhibitor) [171], CPA-1, CPA-3 and CPA-7 [172] to prevent DNA binding; cyclin D1 promoter inhibitor inS3-54A18 [173]; MMP-2 promoter inhibitor salidroside [174]; and pyrimethamine-based STAT3 DNA domain binding (DBD) inhibitor 11d (Figure 10) [175]. However, none of the STAT3 pathway inhibitors disclosed to date have been approved by the FDA.

### 3.4. Arachidonic Acid Metabolism Pathway and Inflammation

**Figure 11:**
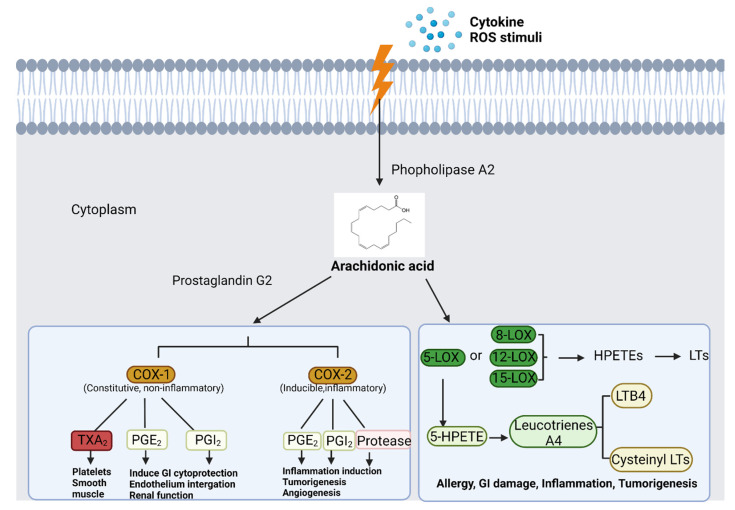
COX and LOX pathways showing the cytokine- and ROS-stimulated production of pro-inflammatory and tumorigenic metabolites.

Arachidonic acid is a precursor of the metabolite prostanoids which are the lipid mediators of inflammatory response (Figure 11). Prostanoids include prostaglandins (PGs), prostacyclin (PGI) and thromboxane (TX). PGs act as signals for cell–cell communications and control inflammation status via intracellular and intercellular signaling. There are various types of PGs with different functions. For example, prostaglandin E2 (PGE_2_) acts as a pro-inflammatory mediator which may trigger pain, swelling, redness and other immune responses in the injured region [176]. Prostacyclin (prostaglandin I2, or PGI) is another prostanoid that prevents platelet formation and attenuates vascular contraction [177]. Recently, PGI has been shown to be an anti-inflammatory mediator which could modulate immune system and attenuate inflammation in tissues [178]. On the other hand, thromboxane (TXA_2_) acts in the opposite direction as it promotes platelet formation [179], vasoconstriction [180] and causes Prinzmetal’s angina [181]. In addition, TXA_2_ could promote inflammation, progression and metastasis in multiple tumors [182].

**Figure 12:**
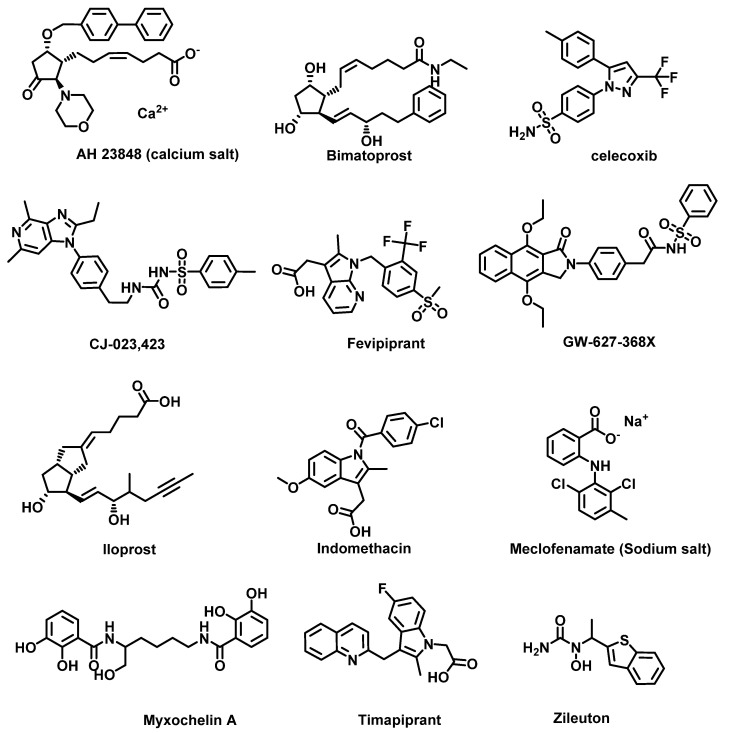
Structures of representative examples of small molecule inhibitors of arachidonic acid pathway.

Drugs such as corticosteroid, which inhibit PGE_2_ receptors, have been found to effectively block the inflammation process. Other PG EP4 receptor antagonists such as GW627368X [183], CJ-023,423 [184] and AH23848 [185] (Figure 12) have shown promising efficacy in blocking the synthesis of PGE_2_ or TXA_2_.

Enzymes, such as cyclooxygenases (COX), which control the metabolism of arachidonic acid (Figure 11), have attracted attention as therapeutic targets. COX families include COX-1, COX-2, COX-3 and COX-IV. In this family, COX-1 and COX-2 are the ones that have been well studied as targets of inflammation and cancer treatment. COX-1 is a constitutively expressed enzyme with critical roles in tissue protection such that it guards the gastrointestinal tract with the synthesis of prostaglandins essential for the maintenance of mucosal integrity [186]. Recently, however, studies have found that COX-1 is over-expressed and highly relevant to the etiology of ovarian cancer. COX-1 intersects with multiple pro-tumorigenic pathways in high-grade serous ovarian cancer [187]. In breast cancer, stromal cells highly overexpress COX-1, which may play significant roles in tumorigenesis [188]. Highly expressed COX-1 in cervical cancer is known to regulate COX-2, PGE2 receptors and angiogenic factors [189]. Therefore, the inhibition of COX-1 may be a chemoprevention strategy for cancers. In contrast, COX-2 is a destructive enzyme that is not active in normal conditions. COX-2 overexpression and high activities are found in injured tissues, fibrotic tissues and tumors. COX-2 is known to be highly active in multiple cancers including breast [190], prostate [191] and liver cancers [192]. COX-2 promotes tumor growth, apoptosis resistance and angiogenesis [193,194,195]. Thus, COX-2 inhibition is a promising drug discovery strategy for anti-inflammation and anti-cancer purposes.

Lipoxygenases (LOXs), a class of iron-containing metalloproteins, are a category of enzymes that regulate the arachidonic acid metabolisms (Figure 11). LOXs catalyze the transformation of arachidonic acid into three types of hydroxyeicosatetraenoic acids (HETEs)—5-HPETE or 5-HETE by 5-LOX, 12-HETEs by 12-LOX and 15-HETEs by 15-LOX. 5-HETE is the precursor of leukotriene LTA_4_, which is converted into lipid mediators LTB_4,_ LTC_4,_ LTD_4,_ LTE_4_ that induce asthma and inflammation [196,197]. Studies have found that 5-HETE and 5-HPETE stimulate the generation of superoxide in human neutrophils, and trigger the ROS stress [198]. The product LTs are complementary pro-inflammatory factors to the PGE_2_. Similarly to COX, 5-LOX contributes to tumorigenesis by directly promoting tumor cell proliferation, growth and survival through the upregulation of LTs [199]. For example, in a colon cancer study, 5-LOX expression was found to be positively correlated with polyp size, intraepithelial neoplasia and adenoma, suggesting that LOX may contribute to the early stage of colon cancer [200]. On the other hand, 12-LOX expression was found to be correlated with advanced stages of prostate cancer [201], and an elevation of urinary levels of 12-HETE has been found in prostate cancer patients [202]. Therefore, LOXs represent another target in the arachidonic acid pathway for the discovery of novel anti-inflammation and anti-cancer agents.

The current strategy for the inhibition of the arachidonic acid metabolism pathway focuses on PG receptor inhibition, PGE_2_ production inhibition, COX inhibition and LOX inhibition. Specifically, PG receptor antagonists (Figure 12) have been widely used for anti-inflammation therapy. For example, timapiprant is a prostanoid receptor 2 (DP_2_) inhibitor which is under a clinical trial for Rhinovirus Challenge in Asthma (NCT02660489) [203]; iloprost is a PG receptor inhibitor used for treatment of pulmonary arterial hypertension (PAH) [204]; fevipiprant is a PG DP_2_ receptor inhibitor currently in a Phase III clinical trial (NCT02555683) for the treatment of asthma [205]; and bimatoprost, a PG analog that acts to prevent the progression of glaucoma, is in use to manage ocular hypertension [206]. The inhibition of PGE_2_ production by non-steroidal anti-inflammatory drugs (NSAIDs) is also effective; however, several early NSAIDs are non-selective COX inhibitors. For example, ibuprofen, indomethacin (Figure 12) and aspirin are NSAIDs with no COX-1/2 selective index [207]. A new generation of NSAIDs showing COX-2 inhibition have been developed. For example, celecoxib (Figure 12) is a selective COX-2 inhibitor [207], which is still in use as an anti-inflammatory drug for the purpose of relieving pain, swelling and rheumatoid arthritis. More recently, LOX inhibitors are attracting more attention for their potential in anti-inflammation and anti-cancer therapy. 5-LOX inhibitors include meclofenamate sodium, zileuton and myxochelins. Meclofenamate sodium effectively suppresses the production of LTD_4_ and attenuates asthma [208]. Zileuton also downregulates several LTs and is also used for managing asthma [209]. Myxochelins/pseudochelins are newly discovered 5-LOX inhibitors that significantly suppress LT production as well [210].

### 3.5. Epigenetic Dysfunction

**Figure 13:**
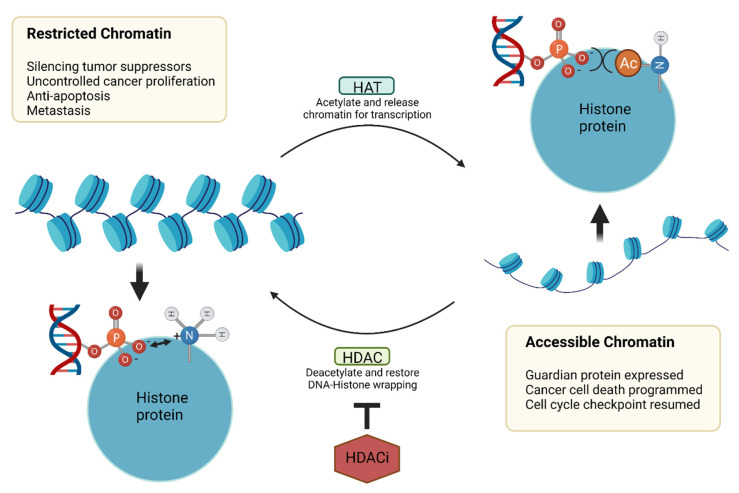
Mechanism of chromatin remodeling by HDACs and HATs.

Histone acetyltransferases (HATs) and deacetylases (HDACs) are key epigenetic enzymes that regulate chromatin dynamics (Figure 13) [211]. HATs acetylate the lysine residue of histones to generate an “open” form of chromatin that is accessible to transcription factors. Nuclear HDACs promote the reverse reaction to generate restricted chromatin. HDACs also deacetylate other non-histone proteins. There are eighteen HDAC isoforms grouped into four classes [212]. Classes I, II and IV are zinc-dependent amidohydrolases, while class III comprises NAD^+^-dependent deacetylases. Class I consists of HDACs 1, 2, 3 and 8, which are mostly located in the nucleus; class IIa comprises of HDACs 4, 5, 7 and 9, which can shuttle between the nucleus and cytoplasm. Class IIb members are HDACs 6 and 10, which are found in cytoplasm, while the only member of class IV is HDAC 11. Class III are sirtuins, consisting of SIRT1-7.

Dysfunctions in HDAC activities could promote cancer, inflammation and immune response. In cancers, HDACs play important roles in upregulating oncogene expressions while restricting the expression of tumor suppressors, resulting in the inhibition of cell death (Figure 13). To facilitate the pro-survival tendency of the tumor microenvironment, HDACs could also upregulate the expressions of genes for angiogenesis, autophagy, metastasis and immune homeostasis [211]. HDACs also contribute to tissue inflammation and fibrosis. For example, the upregulation of HDAC1 and 2 activity stimulates the TGF-β pathway as well as induces EMT [213] and ECM overproduction; and promotes fibroblast–myofibroblast transformation as well as cell migration. Additionally, HDACs promote the expression of cytokines such as TNF-α, IL-6 and IL-1β. HDAC3 specifically recruits the NF-κB/p65 signal through the epigenetic regulation of IL-1 expression and thus TNF-α induced inflammation [214,215]. HDAC1 and HDAC2 are evidenced to be responsible for the positive regulation of IL-6- and STAT3-induced gene expression [216].

Aberrant HDAC activities have also been implicated in fibrosis including in liver cirrhosis, cardiac fibrosis, pulmonary fibrosis, renal fibrosis and other inflammation diseases [217]. HDAC inhibitors (HDACi) MS-275 and TSA suppress the TGF-β-mediated MAPK and PI3K pathway inflammation signals by the demonstrable downregulation of biomarkers of p-ERK and p-Akt [218]. TSA also attenuates tissue inflammation and prevents further damage [219,220]. HDAC inhibition also regulates the STAT pathway by the hyperacetylation of STAT proteins [221]. Specifically, the acetylation of STAT3 accelerates the STAT3 translocation towards the mitochondria where the binding of acetylated STAT3 to the E1 subunit of pyruvate dehydrogenase (PDH) stimulates the conversion of pyruvate into acetyl-CoA to increase metabolic flux through the TCA cycle that will consequently stimulate energy production via oxidative phosphorylation [221]. HDAC inhibition can also suppress tissue inflammation through the inhibition of COX activation and PGE production [222].

**Figure 14:**
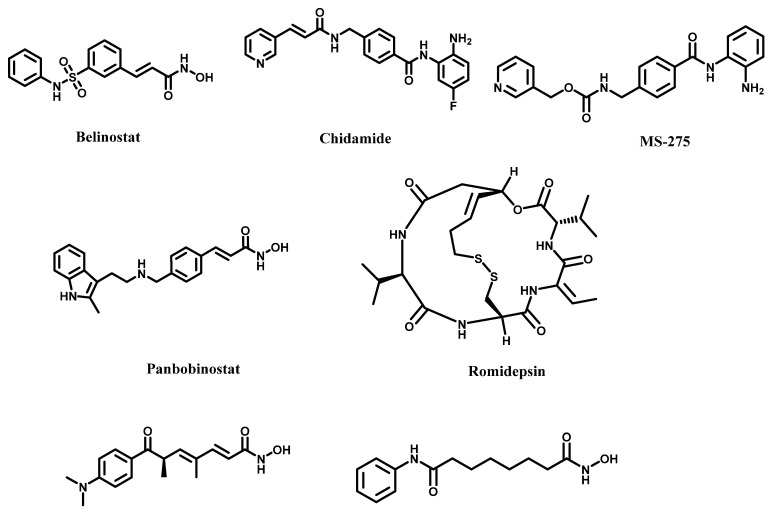
Structures of representative examples of HDAC inhibitors.

The major interest in HDAC dysregulation has been for its implications in carcinogenesis. There is solid evidence to show that HDACs play an essential role in epigenetic transformations that ultimately sustain tumor viability and invasiveness [223]. It has been found that HDACs 1 and 6 could control the invasiveness of clear cell renal cell carcinoma (ccRCC). HDAC 6 is capable of inhibiting p53 to promote tumor progression in HCC. The FDA and other non-US regulatory authorities have to date approved five HDACi (Figure 14) to treat hematological malignancies. Vorinostat (SAHA) is approved for cutaneous T-cell lymphoma [224]. Belinostat, chidamide and romidepsin have been approved for peripheral T-cell lymphoma [225,226,227], while panobinostat is approved for multiple myeloma [228]. The standard pharmacophoric model of zinc-dependent HDACi consists of a zinc-binding group (ZBG), a hydrophobic linker and a recognition cap group. Common ZBGs include carboxylic acid, hydroxamic acid, thiol, trifluoroketones and benzamide [229]. To address the pharmacokinetic and pharmacodynamic issues plaguing early ZBGs, there has been sustained interest in identifying novel ZBGs [230]. In this regard, several non-hydroxamic acid moieties, including those found in naturally occurring flavonoids (genistein, chrysin, etc.), have been reported as HDACi with novel ZBGs [231].

Other researchers have also reported novel synthetic ZGs [229,232,233,234,235,236]. These new ZBGs are promising templates for the design of a new generation of HDACi that may possess targeted anti-inflammation and anti-cancer activities with reduced systemic toxicity.

## 4. Current Medical Challenges and Novel Solutions for Targeting Inflammation Pathways

### 4.1. TGF-β Pathway

The inhibition of the TGF-β pathway as an anti-inflammation and anti-cancer treatment strategy has encountered several challenges including overt toxicity, off-target effects and low in vivo efficacy. The toxicity that results from TGF-β pathway inhibition is due to the vital multifaceted roles of TGF-β pathway in normal biology. The TGF-β pathway maintains tissue homeostasis and repair [237]. In normal tissue and organs, TGF-β promotes tissue regeneration and repair by recruiting stem/progenitor cells [238]. The TGF-β pathway has also been shown to induce anti-oxidative effect via the suppression of COX-2 activities in lung cancer cells [239]. Thus, the inhibition of TGF-β may cause adverse effects [240]. Five drug candidates targeting the upstream of the TGF-β pathway—the integrin inhibition—have been studied in the clinic for cancer treatment. Despite the promising data from preclinical animal models, the clinical outcomes have not been encouraging. Two trials (NCT01122888, NCT02337309) were terminated due to a lack positive of outcome and the results from three trials (NCT00721669, NCT00284817, NCT00635193) have not been disclosed to the public to date [241].

As described in Section 3.1 above, the TGF-β pathway is regulated by several other pathways whose individual or collective inhibition could overcome the challenges noted with integrin inhibition. Pleiotropic small molecules that inhibit cohorts of TGF-β signaling mediators have been investigated. PFD, a notable example that suppresses the TGF-β pathway through MAPK inhibition, has been approved by the FDA for the management of IPF [122,123]. The potential of PFD as an anti-tumor agent is under active investigation [124]. However, PFD’s systematic toxicity and low bioavailability have limited its efficacy in IPF treatment. PFD only slows long-term IPF progression with 2.47 years life expectancy extension with no evidence of stopping the disease progression [242].

Structure–activity relationship (SAR) studies may be employed to further improve the therapeutic indices of pleiotropic agents. Recent computational screening (CDOCKER) on pyrrolotriazine-like pharmacophore revealed the contribution of this pharmacophore in selective TGF-β inhibition [243]. Pyrrolotriazine-like pharmacophore is conserved in known TGF-β receptor (TβRI) inhibitors including SB431542 [244], LY2109761 [245] and galunisertib [246], indicating that the TβRI ALK-5 preferred this moiety. However, none of the candidate TGF-β inhibitors have succeeded as anti-cancer agents in clinical trials. Nevertheless, TGF-β inhibitors remain of high value in fibrosis and cancer therapy. The previously discovered agents could aid new SAR studies to later generation agents with improved safety profiles. To improve the targeting of disease sites, therapies including antibody inhibitors and antisense are rising as promising strategies for targeting specific proteins. The reported TGF-β targeting therapies include antibody drug GC1008 as a ligand neutralizer, antisense such as AP12009 and AP11014 (translation inhibitors that bind to the mRNAs of TGF-β2-related genes.

Additionally, pleiotropic drugs may not be all that bad as some TGF-related inflammatory functions could be restored by other pathways such as the MAPK pathway and the PI3K pathway. PFD could be a good choice in this aspect. However, PFD lacks tissue or cell-type selectivity, which significantly lower its therapeutic index. To minimize the systematic exposure of TGF-β inhibitors such as PFD, lung-targeted delivery using a nanoparticle-based vehicle has been investigated. Poly(lactide-co-glycolide) nanoparticles-formulated PFD has demonstrated significantly improved efficacy in a murine model of IPF [237]. Another potential future improvement to PFD is the incorporation of tissue-selective small molecule-based delivery vehicles or pro-moieties that may function to target the PFD into the desired tissue. While TGF-β inhibitors prodrugs have appeared in the literature [247], conjugation to tissue-accumulating templates has been well studied [248]. These strategies provide a great opportunity to reduce the systematic toxicity of TGF-β inhibitors.

### 4.2. Challenges with NF-κB Pathway Inhibition

The NF-κB pathway is important for both normal cell and diseased cell regulation and the gain of function mutations within this pathway is relatively rare [249,250]. This has contributed to the low therapeutic indices of NF-κB-targeting agents. In addition, not all cancer cells are solely dependent on NF-κB for survival, which makes most NF-κB inhibitors only suppress cancer cell proliferation without causing cancer cell death. Paradoxically, NF-κB inhibition may even augment the invasiveness of certain cancer cells as it has been observed that ovarian cancer cells could become more invasive with NF-κB inhibition [251]. Additionally, NF-κB may play a very important role in immunity, and NF-κB inhibitors could induce the impairment of NF-κB-dependent immune response and inflammation [249].

Instead of direct inhibition, Nrf-2 activation is an alternative approach to achieve NF-κB inhibition [252]. Activated Nrf-2 induces the transcription of anti-ROS genes such as HO-1, SOD, NQO1, catalase and other anti-oxidant proteins. These proteins effectively suppress the NF-κB signal and attenuate inflammation progression. Most importantly, the Nrf-2 activation does not impair the normal NF-κB pathway in the cell, and so effectively reduces inflammation with reduced adverse effects. Nrf-2 activation could be achieved through the inhibition of Kelch-like ECH-associated protein 1 (KEAP1). To date, known KEAP1 inhibitors, such as dimethyl fumarate (DMF), are electrophilic compounds that form covalent bonds with its cysteine 288 and 289 [147,253,254]. DMF has been approved for the treatment of relapsing multiple sclerosis and psoriasis while clinical trials evaluating its potential application in other inflammatory diseases and cancer are ongoing [255]. Piperic acid (PPA) and its analog, piperine, are another example of KEAP1 inhibitors whose mechanisms of action have not been firmly established [256]. The PPA and DMF may work similarly to inhibit KEAP1 activity. However, DMF and PPA lack tissue targeting and are widely distributed in the human body. The systemic distribution could be responsible for the adverse effects such as abdominal pain, flushing, diarrhea, nausea and etc., which DMF has elicited in the clinic [257].

Therapeutic indices of the direct inhibitors of NF-κB could be improved by employing strategies that enable direct targeting to tumor-sites and/or inflamed tissues. In this regard, the potential of nanoparticles as delivery vehicles has been investigated. Peptidic nanoparticles complexed to NF-κB siRNA have been shown to penetrate human cartilage to deliver their siRNA cargo to elicit significant NF-κB inhibition, resulting in the protection of chondrocytes from NF-κB-induced death [258]. However, metal nanoparticles, such as gold/silver materials, have contradicting effects on NF-κB function. Some have reported that gold NPs (Au-NPs) reversibly induced NF-κB activation in B-cell lymphocytes and caused inflammatory side effects [259]—while others have shown that gold NPs could suppress NF-κB pathway inflammation through the downregulation of COX-2 expression in a rheumatoid arthritis model [260]. The difference might be based on cell type and tissues. Additionally, antibody drug conjugation (ADC) could benefit the cell-type/tumor selectivity of small molecules. A recent report has found that an anti-p65 antibody conjugated with TAT resulted in an outstanding translocation blocker of the NF-κB pathway [261]. Such a design could lower the systematic exposure of drugs and thus increase the specificity of NF-κB inhibition.

### 4.3. Challenges with COX Inhibition

Recent efforts to achieve COX inhibition have mainly focused on selective COX-2 inhibition. Celecoxib, a lead FDA-approved COX-2 inhibitor, is being investigated in clinical trials for the treatment of several cancer types including head and neck cancer (NCT04162873), bladder cancer (NCT02885974), TNBC (NCT04081389) and malignant pleural mesothelioma (NCT03710876); as well as for antiangiogenic therapy to treat medulloblastoma, ependymoma ATRT (NCT01356290) and others. In previous trials, celecoxib has been studied in combination therapy with other chemotherapy agents. COX-2 overexpression had been found to increase cancer cell drug resistance to chemotherapy, and the combination of the COX-2 inhibitor may induce drug sensitivity instead [262,263]. However, in previous clinical trials, HER2 positive breast cancer patients that were treated with trastuzumab along with celecoxib did not experience improved efficacy [264]. In a colorectal cancer chemoprevention study, cardiovascular risks (heart failure) was observed in patients taking a high daily dosage of celecoxib for a period of 3 years [265]. Another study has also noticed celecoxib’s cardiovascular risks [266]. Valdecoxib, another selective COX-2 inhibitor, was voluntarily withdrawn due to cardiovascular concerns [267].

Although the role of COX-2 in malignant transformations is well established, COX-1 also plays a leading role in tumorigenesis (see Section 3.4 for details). Unfortunately, truly selective COX-1 inhibitors are few and remain to be well studied to decipher the roles of COX-1 in the pathophysiology of cancers [268]. Although several traditional NSAIDs have COX-1 inhibition activities, they are beleaguered by gastrointestinal toxicity which is yet to be resolved to date [269]. Strategies that reduce NSAIDs-induced gastrointestinal toxicity could improve NSAIDs’ utility as anti-inflammatory agents and expand their therapeutic applications to oncology.

### 4.4. Challenges with STAT3 Inhibition and Solution

The inhibition of multiple effectors, including JAK kinase, SH2 Domain and DNA binding domain have been investigated as a strategy to inhibit the STAT3 pathway [270]. However, the FDA has approved none of the lead agents targeting these effectors for cancer treatment. The failure of current STAT3 inhibitors is largely due to toxicity and a lack of specificity for STAT3. Despite this frustration, STAT3 still remains a valuable target for the discovery of anti-cancer drugs. Since STAT3 is a major protein that maintains cell proliferation and survival, a non-specific STAT3 inhibitor could induce intolerable systematic side effects. The integration of disease site selectivity into the design of a new generation of STAT3 inhibitors may be necessary. For example, nanoparticles (NPs), due to an enhanced permeability and retention (EPR) effect, may enhance STAT3 inhibitor tumor targeting. A recent study showed that anti-CD38 antibodies decorated NPs loaded with the STAT3 inhibitor S3I-1757 which possess in vitro STAT3 inhibition and elicit significant improvement in tumor suppression in a xenograft mice model [271]. STAT3 inhibitors may also be good candidates for combination therapy with other anti-cancer agents. For example, a combination therapy of the STAT3 inhibitor (STX-0119) and GLI1/tGLI1 inhibitor has been investigated as a breast cancer treatment strategy [272]. GLI1/tGLI1 is overexpressed and it is an important protein for maintaining the proliferation and anti-apoptosis of breast cancer stem cell self-renewal. This combination treatment showed a significant suppression of an angiogenesis and anti-apoptosis effect in tumor cells with the downregulation of STAT3 Bcl-2 families and VEGFR mRNA. In vivo, this combination showed an improvement in the suppression of tumor growth with no significant toxicity to mice at the tested dose. In contrast, the combination of STAT3 inhibitors with PD-L1 inhibitors has been shown to promote PD-L1 expression and suppress the immune response instead, which leads to the reduction in the anti-tumor effect of the anti-PD-L1 antibody [273,274]. This is a cautionary observation which suggests that the partners of STAT3 inhibitors in combination therapy must be carefully selected to ensure optimum therapeutic benefits.

Recently, STAT3 pathway inhibition has been shown to be well suited for anti-inflammation and immune regulation. JAK inhibition is an increasingly popular strategy in this regard [275]. Olumiant (baricitinib), a JAK1/2 inhibitor developed by Incyte, has been used for the treatment of rheumatoid arthritis, a type I inflammatory disease. Other JAK inhibitors, such as ruxolitinib, upadacitinib and abrocitinib, are also being investigated for the treatment of eczema (atopic dermatitis) [276].

### 4.5. Challenges with HDAC Inhibition

Although HDAC inhibition has been clinically validated for the treatment of hematological malignancies, it has so far not been effective against solid tumors. The reason for the lack of efficacy of HDACi against solid tumors is not fully understood. However, the fact that most HDACi do not accumulate within the tumor interstitium at therapeutically efficacious concentrations is a major cause [223]. Additionally, HDAC inhibition elicits a pleiotropic phenotype, primarily due to their non-selective inhibition of various HDAC isoforms and possibly other non-HDAC targets. This non-selectivity causes reduced in vivo potency and toxic side effects. Regardless, the reactivation of tumor-suppressor genes by HDACi is still currently being pursued as a potentially broad cancer treatment option [277].

Because several cancer types exhibit dysfunctions in the regulation of various HDAC isoforms, a combination of the selective inhibition of HDAC isoforms relevant to the disease etiology and disease-site targeting could further enhance the utility of HDACi in cancer therapy [211]. For example, the overexpression of class I HDACs 1, 2 and 3; and class II HDACs 5 and 6 has been shown to be crucial to the viability of liver cancer cells, including HCC cell lines [278]. Earlier studies have explored the potential of the intratumoral administration of a short-chain fatty acid HDACi (4-phenylbutyrate) and a HDACi pro-drug (hyaluronate-butyrate) designed to target membrane receptor CD44 on liver sinusoidal epithelial cells [279,280]. A more recent study disclosed and characterized macrolide-based liver-tissue accumulating selective HDAC 1 inhibitors [281]. These studies revealed that these delivery strategies facilitated potent HDAC inhibition at the disease site, which resulted in a significant repression of HCC tumor growth and metastatic spread. These literature precedents suggest that HDACi liver tissue accumulation could be a promising strategy to expand the therapeutic applications of HDACi to include solid tumors such as liver cancer. The combination of HDACi with other anti-cancer agents is another strategy that is currently being investigated to overcome the shortcomings seen in HDACi monotherapy. Currently, ongoing preclinical and clinical evaluations include combination therapy regimens of HDACi with drugs that target DNA repair pathways, topoisomerase inhibitors, platinum-based chemotherapeutics, tyrosine kinase pathway inhibitors, proteasome inhibitors, hormone therapy, immunotherapy and radiotherapy [282]. Although none of these combination therapy studies has furnished approved agents, the results that have been made available in the literature are encouraging. One important highlight is the observation that HDAC inhibition could improve cancer cells’ immune response via the regulation of the expressions of tumor antigens, including CD40, CD80, CD86, MHC, ICAM-1 and MICA/B, which drive the elimination of cancer cell proliferation through the immune system [283,284,285,286]. In addition, HDACi showed the upregulation of the expression of PD-L1 in melanoma cells and potentially augment the response to immunotherapy in patients that are resistant to anti-PD1 antibody therapy [287].

Relative to their potential in oncological applications, the repurposing of HDACi for anti-inflammation and anti-fibrosis therapies has not been widely studied. As stated in Section 3.5, HDACi could regulate several inflammation signals including EMT and control cytokine expression to promote the resolution of tissue inflammation. HDACi could reduce the progression of collagen-induced arthritis in murine models by inhibiting the IL-1 and IL-6 expression by 95% [288]. Moreover, HDACi could regulate the transcription of inflammatory transcriptional factors such as NF-κB and STAT3, which may affect cell survival and prevent inflammation progression. Encouraging data have been disclosed about the ameliorative effects of HDACi on cardiac and pulmonary fibrosis. HDACi showed both a therapeutic and preventive function in two types of fibrosis in animal models [289]. However, it has been observed that HDACi may also promote some inflammatory cytokines because acetylation on non-histone proteins could suppress the anti-inflammatory proteins [290]. For example, TSA, a pan-HDACi, induced the upregulation of IL-8 (an inflammatory inducer) but repressed IL-12 (an activator of cytotoxic immune cells) in the BEAS-2B [291]. Moreover, it has also been observed that TSA could suppress pro-inflammatory gene mRNAs related to the TLR-4 pathway (IL-1, IL-10, TNF-α, IKKε and iNOS) in a murine macrophage, while it significantly upregulates the expression of COX-2 [292]. The same study also analyzed the functions of different HDACs and concluded that different HDACs may contribute to different functions in a macrophage and cause both pro- and anti-inflammatory effects [292]. The fact that HDACi may elicit both anti- and pro-inflammatory effects could constitute a major challenge to the repurposing of HDACi for anti-inflammation and anti-fibrosis therapies. Despite these unresolved challenges, HDACi remain high value agents for the treatment of inflammation, fibrosis and cancer. There is a need for a more mechanistic understanding of the roles of HDACi in these diseases to bring the therapeutic potential of HDACi to fruition.

## 5. Conclusions and Future Outlook

Inflammation is strongly linked to several serious chronic diseases that remain to be effectively managed in the clinic. Death rates from several of these diseases, including IPF, liver cirrhosis and gastric inflammation are very high. There is copious evidence that implicates several of these diseases in malignant transformations. Key pro-inflammatory signals, including the TGF-β pathway, NF-κB, STAT pathway, arachidonic acid metabolism and epigenetic regulations that sustain these diseases also play essential roles in tumorigenesis. Although we now have an in-depth understanding of the mechanism of each pathway and their roles in inflammation, several pharmacological agents that are currently in use for the treatment of chronic inflammatory diseases and tumors are sub-optimal. One major reason is that most of these agents are systemic agents that are plagued with overt toxicity and poor distribution at disease sites.

In this review, we thoroughly explored the connection between chronic inflammatory diseases and tumor. We also described how drug delivery systems, combination therapy, the integration of tissue-targeted and/or pathway selective strategies could overcome the challenges of the current agents for managing and/or treating chronic inflammatory diseases and tumors. We expect that the understanding that chronic inflammatory diseases and tumors are driven by the dysregulation of multiple pathways, which are essential for normal biology, will influence the design of a new generation of therapies for managing these diseases. The therapeutic indices of the currently available agents could be improved using delivery strategies that enable the disease-site accumulation of drugs as standalone agents and/or part of combination therapy regimens. It is conceivable that the incorporation of several current systemic agents into nano-formulations with or without homing ligands could transform them into “targeted agents”. Pleiotropic agents, which are often given less priority but may be ideal for multi-pathway dysfunction diseases such as chronic inflammation and cancers, will immensely benefit from such a disease-site delivery strategy.

Another future direction for drug development is to re-evaluate the roles of each pathway in specific diseases. For example, for decades, COX-2 was regarded as the major inflammatory and tumorigenic protein because its upregulation was seen in diseases such as colorectal cancer and lung asthma. However, recent observations have revealed that COX-2 is correlated to COX-1 and COX-1 is possibly causing inflammation in some cases. For example, COX-1, overexpressed in cervix cancer, regulates COX-2 and PGE_2_ level [189]. In fact, COX-1, over-expressed in ovarian cancer [187,293] is now suggested to be a prognostic biomarker [294]. Another example can be found in another study that contradicted the roles of STAT1 in cancer. STAT1 is commonly regarded as a tumor suppressor which promotes the apoptosis of cancer cells and activates immunity [295]. However, STAT1 could also act as a double-edged sword in inflammation and cancers. For example, STAT1 is implicated in the development of radiotherapy resistance. STAT1 inhibitors seem to provide benefits to overcome radiotherapy resistance [296]. Additionally, the overexpression of STAT1 in both malignant breast and ovarian cancer is likely to induce tumor proliferation and other oncogenic activity [297,298]. Therefore, more studies on the relationship between pathways and specific disease are still vital to the successful development of efficacious therapies.

In light of the role of the host immune system in the development of inflammation and tumorigenesis, targeting the immune cells promoting such pathogenesis may hold significant therapeutic benefit. Historically, the methods used to identify immune cell types in a tissue have relied on immunohistochemistry or flow cytometry whose results can be impacted by tissue degradation and sampling errors. Single-cell RNA sequencing data enable the identification of cell types based on their gene expression profile. A computational method such as cell-type identification by estimating relative subsets of RNA transcripts (CIBERSORT) can be applied to the single-cell RNA sequencing data to enable the identification of the relative proportion of 22 different immune cell types in the tissue or tumor microenvironment [299]. With the ensuing knowledge of the immune cell types present in the microenvironment, targeting the cell types promoting the pathogenesis may not only identify novel targets but also yield potentially safer therapies directed towards specific cell types rather than pathways which may be critical for cellular homeostasis.

## Figures and Tables

**Table 1:** Inflammation-associated cancer types [7,85,87].

Inflammation Conditions	Cancern Type	Inducer
Asbestosis	Lung carcinoma	Silica
Chronic bronchitis	Lung carcinoma	Silica
IPF	Lung carcinoma	Unclear
Tuberculosis	Lung carcinoma	Mycobacterium tuberculosis
Liver cirrhosis	HCC	Hepatitis infection, alcoholic, genetic
IBD, Crohn’s disease, chronic ulcerative colitis	Colorectal cancer	Gut pathogens
Chronic gastric inflammation	Gastric cancer	*Helicobacter pylori*
Reflux oesophagitis, Barrett’s oesophagus	Oesophageal carcinoma	Gastric acids
Skin inflammation	Melanoma	UV light
Chronic pancreatitis, hereditary pancreatitis	Pancreatic carcinoma	Alcohol, gene mutation
Schistosomiasis	Bladder carcinoma	Gram-uropathogens
Cervicitis	Cervical cancer	Human papilloma virus
Chronic prostatitis	Prostate cancer	Bacterial infection
Sialadenitis	Salivary gland carcinoma	Bacterial infection
Sjögrensyndrome, Hashimoto’s thyroiditis	MALT lymphoma	unclear
Gingivitis, lichen planus	Oral squamous cell carcinoma	Bacterial infection
Chronic cholecystitis	Gall bladder cancer	Bacteria, gall bladder stones

**Table 2:** Possible STAT heterodimers and their inflammation and anti-inflammation activators [159].

STAT Protein Types	Stimulators (Inflammation)	Stimulators (Anti-Inflammation)	Heterodimerization
STAT1	Type I IFN Type II IFN IL-6	IL-10 IL-27 IL-35	STAT2 STAT3 STAT4
STAT2	Type I IFN		STAT1 STAT6
STAT3	IL-2 IL-5 IL-6 IL-23 MCSF GCSF Type-II IFN	IL-10 IL-27	STAT1 STAT4 STAT5
STAT4	IL-12 IL-23	IL-35	STAT1 STAT3
STAT5	IL-2, IL-9, IL-15 IL-21 MCSF GCSF		STAT3
STAT6	Type I IFN IL-3, IL-4, IL-13		STAT2
